# Hybrid island-and-sea approach for corrosion protection of Si photocathode in neutral-pH water splitting

**DOI:** 10.1038/s41598-025-30589-y

**Published:** 2025-12-03

**Authors:** Magzhan Amze, Asset Aliyev, Yerbolat Magazov, Nurxat Nuraje, Kadyrzhan Dikhanbayev, Erkin Shabdan

**Affiliations:** 1 Renewable Energy Lab, National Laboratory Astana, Nazarbayev University, Astana, 010000 Kazakhstan; 2 Department of Chemical and Materials Engineering, School of Engineering and Digital Sciences, Nazarbayev University, Astana, 010000 Kazakhstan; 3 Faculty of Physics and Technology, Al-Farabi Kazakh National University, Almaty, 050040 Kazakhstan; 4 School of Intelligent Systems, Astana IT University, Astana, 010000 Kazakhstan

**Keywords:** Chemistry, Energy science and technology, Materials science

## Abstract

Photoelectrochemical (PEC) water splitting is a promising way for converting solar energy into green hydrogen, yet the long-term stability of the studied photoelectrodes remains a main challenge. Therefore, corrosion protection of photocathode and photoanode materials has attracted attention. Here, we demonstrate a simple yet effective “island-and-sea” strategy to enhance the stability of silicon-based photocathodes in neutral media water splitting. Platinum nanoparticles (“islands”) deposited on Si facilitate efficient charge transfer, whereas the remaining surface is passivated with a hydrophobic 1-octadecyl (OD) self-assembled monolayer (“sea”) that acts as a corrosion-resistant barrier. This organic-protective layer allows stable PEC operation without altering the semiconductor’s band structure and complex fabrication steps. The Si/Pt + OD structure maintains a stable photocurrent over 6 h, compared to a 50% decline after 3 h of operation for the unprotected Si/Pt photocathode. Since neutral electrolytes like seawater are readily available and inexpensive natural resources on the planet, the “island-and-sea” approach applicable for stable operation at neutral pH becomes crucial for real-world applications.

**Supplementary Information:**

The online version contains supplementary material available at 10.1038/s41598-025-30589-y.

## Introduction

Water splitting under sun irradiation utilises abundant sunlight energy to produce high-energy-density hydrogen fuel. Honda et al. were the first to use semiconductors to split water under light in an electrochemical cell in 1972^1^. In the last half-century, many photoelectrode materials have been investigated, but photoelectrochemical (PEC) water splitting systems still lack durability, preventing them from being used for commercial energy production^2^. 10,000 h of stable operation of photoelectrodes without losing their efficiency during water splitting is stated to be possible^3^. In light of this, developing novel methods of corrosion protection of PEC electrodes that ensure prolonged stable operation has become one of the holy grails of materials science.

Silicon is an attractive material for PEC water splitting because it absorbs a wide range of the solar spectrum, is earth-abundant, and its production technology is well established^4–7^. However, silicon is not stable in solar hydrogen evolution conditions. Pourbaix diagrams show that at pH 7 under 0.0 V vs. RHE, Si dissolves into H_4_SiO_4_ (aq), H_2_SiO_3_ (aq), SiO_2_ (aq)^8^. This limits the use of silicon photoelectrodes for solar water splitting. Conventionally, researchers attempted to solve this problem by creating buried junctions with layers of corrosion-resistant oxides like TiO_2_^9^, Al_2_O_3_^10,11^, and SrTiO_3_^12^. This method is widespread and used to create protective layers on the surface of different metal oxides, too. However, these protective layers require the use of expensive and hard-to-upscale methods like epitaxial growth and ALD, and the protective layer itself changes the band structure of the electrode.

Alternatively, researchers proposed to use organic protective coatings. Wu et al.^13^ propose to use alkyl silane to increase the hydrophobicity of the surface and take advantage of hydrogen gas accumulation on the surface to separate and protect the photoelectrode from the electrolyte. However, in this method, the silane layer does not totally cover the surface, and there are micron-scale patches still exposed to the electrolyte. Zhang et al.^14^ propose another solution, where a covalent triazine framework is used to create both protective and energetically favourable junctions on an extremely unstable Cu_2_O photocathode and achieve 150 h of operation with a slight efficiency decrease. These examples show that the organic protection approach can also result in prolonged stability in PEC systems.

Durable, corrosion-resistant photoelectrodes are vital for scalable PEC hydrogen generation, particularly under neutral-pH conditions^15–17^. For example, potentiostatic photoactivation of BiVO_4_ yields a cocatalyst-free anode with passivated surface states and oxygen vacancies, achieving record photocurrents (≈ 4.6 mA cm^[- 2^ at 1.23 V vs. RHE) and exhibiting a “self-healing” stability over > 100 h^15^. Similarly, in situ formation of a Ni–B (nickel–borate) layer in the electrolyte suppresses Ni dissolution and charge recombination, enabling a BiVO_4_ electrode to operate stably for over 600 h in a neutral electrolyte while minimizing photocorrosion^16^. These protective strategies reflect broader trends: for instance, a chloride-mediated NbClOx/BiVO_4_ photoanode demonstrated > 500 h stability under one-sun illumination in seawater^18^, and a large-area bias-free PEC device maintained performance for 24 h without degradation^19^. Additionally, advanced catalyst architectures have boosted intrinsic efficiency – for example, Ru–P diatomic sites on Fe_2_O_3_ achieved 4.55 mA cm^[- 2^ at 1.23 V vs. RHE with a 0.58 V vs. RHE onset – showing that high efficiency can be attained without sacrificing stability^20^. Together, these advances underscore that combining corrosion-resistant interfaces and robust catalyst design can yield PEC photocathodes that are both highly efficient and long-lived under neutral conditions^15,16^.

Therefore, herein, we propose an “island-and-sea” approach to protect photoelectrodes during water splitting under neutral pH conditions (~ pH 7). In this approach, electrocatalyst particles like Pt particles are deposited on the surface of a Si photoelectrode and serve as water splitting reaction centres (“island”). The areas not covered with Pt nanoparticles are insulated with a hydrophobic self-assembled monolayer (SAM) (“sea”). In this way, the electrode is separated from the electrolyte by the SAM and at the same time, the charge transfer is ensured through the Pt nanoparticle electrocatalyst. This initial proof of the “island-and-sea” concept in neutral electrolytes paves the way for the development of organic self-assembled protective monolayers for large-scale hydrogen production.

## Results and discussion

**Fig. 1:**
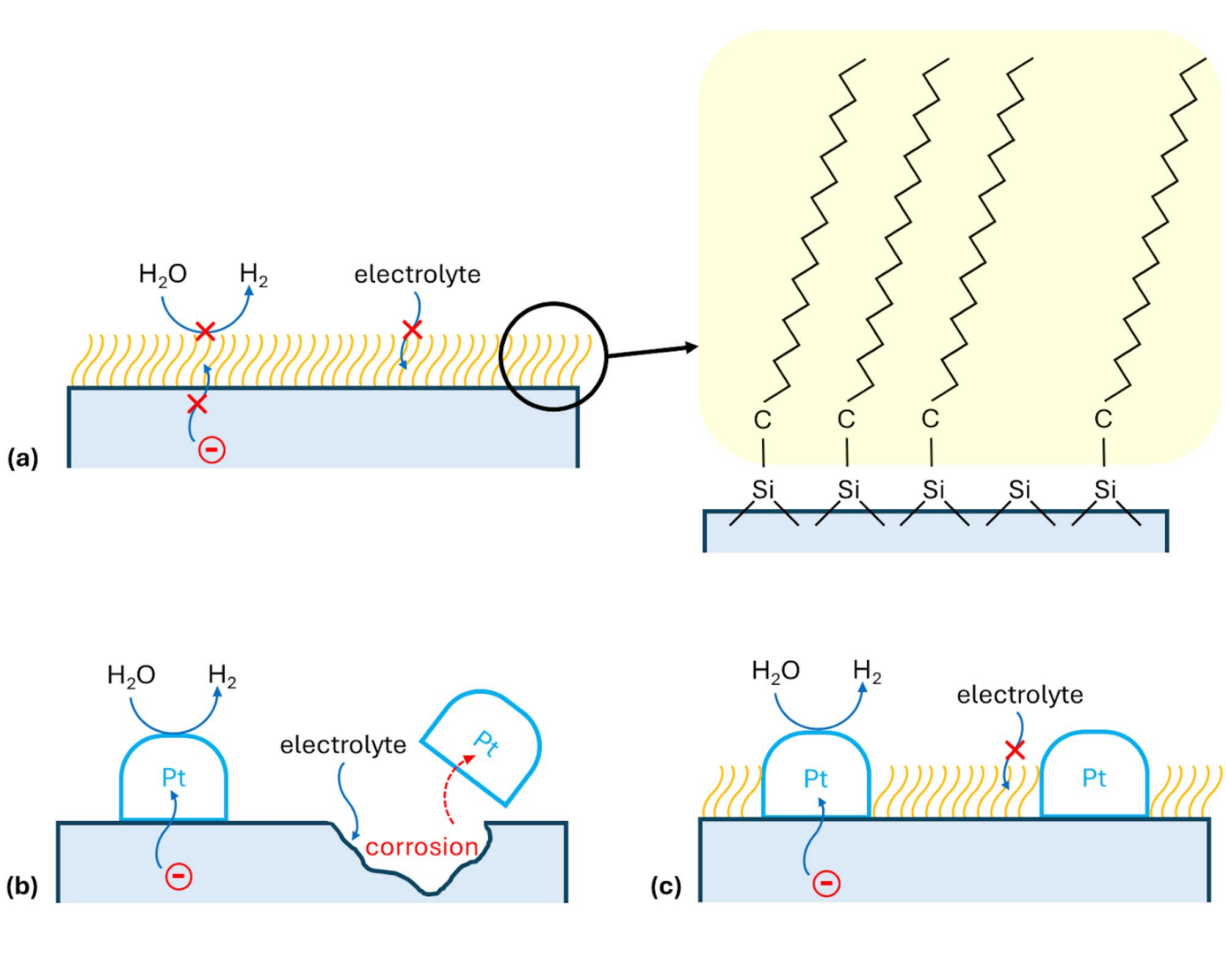
Schematic illustration of an “island-and-sea” approach to protect photoelectrodes during the neutral medium water splitting process. (**a**) c-Si with a hydrophobic OD SAM. (**b**) Corrosion mechanism of c-Si/Pt during water splitting. (**c**) Water splitting through Pt nanoparticles (“island”) over c-Si protected by OD SAM (“sea”).

We present an effective “island-and-sea” strategy (Fig. 1) for protecting photoelectrodes during neutral-pH photoelectrochemical (PEC) water splitting. In this method, electrocatalyst particles such as platinum nanoparticles act as active sites for the water splitting reaction—the “islands”, whereas the remaining surface area, coated with a hydrophobic self-assembled monolayer (SAM), forms the “sea”. This design effectively shields the electrode from direct contact with the electrolyte while still allowing efficient charge transfer through the Pt catalyst particles. One of the advantages of this approach is that the hydrophobic SAM layer formation does not require expensive technologies like ALD or epitaxial growth. It is achieved via solution-based chemical methods. Moreover, the hydrophobic SAM layer does not interfere with the band structure of the semiconductor the way inorganic protective layers do. Considering these advantages, the organic SAM-based “island-and-sea” approach can be an attractive alternative method of corrosion passivation in neutral medium water splitting photoelectrodes.

**Fig. 2:**
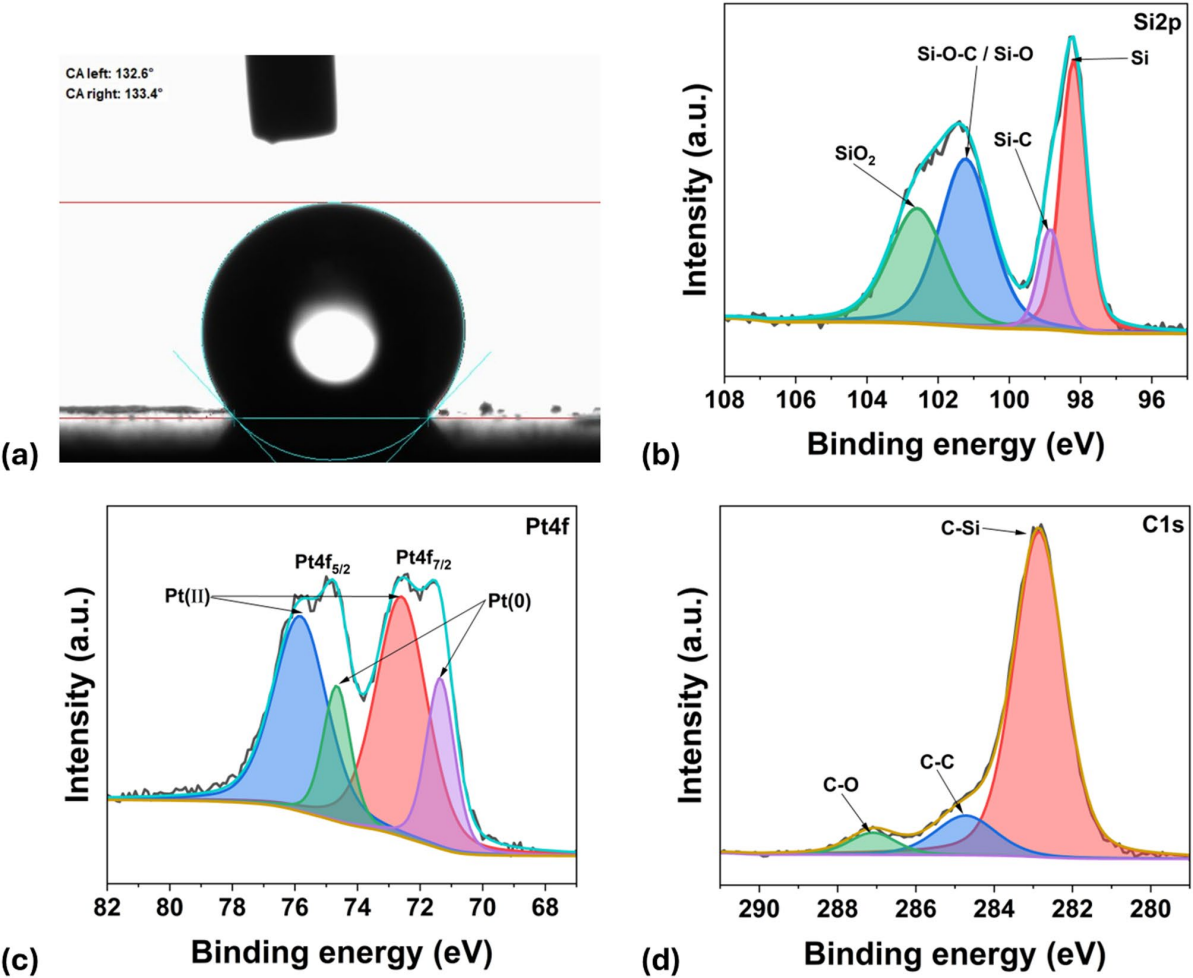
Structural characterisation of Si/Pt + OD. (**a**) Contact angle measurement of the photoelectrode. XPS results for (**b**) Si 2p, (**c**) Pt 4f, and (**d**) C 1 s spectra.

1-Octadecyl SAM is found to be one of the most hydrolytically stable SAMs on Si. 1-octadecene reacts with Si under UV or elevated temperature and forms an octadecyl self-assembled monolayer (SAM), which is chemically stable due to the formation of Si-C covalent bonds, which withstand both acidic and alkaline environments^21^. Contact angle measurement of pn-Si/Pt samples shows that after the reaction with 1-octadecene, the surface of the electrodes becomes hydrophobic (~ 132°) (Fig. 2a). This is an indication that 1-octadecene formed a protective layer on the surface of the electrode. However, despite that, totally covering the surface with the SAM will protect it from corrosion, but it will not allow charge transfer between the electrode and the electrolyte. The charge transfer through the interface is achieved by deposition of Pt nanoparticles. In Figure S1, the SEM image shows the formation of Pt particles with a size of 30–50 nm throughout the surface of pn-Si. These particles allow charges to drive from pn-Si to the hydrogen evolution reaction and are an essential part of the “island-and-sea” scheme.

XPS analysis was employed to confirm the successful formation of the protective monolayer and the presence of Pt nanoparticles. The Si 2p spectrum showed a signal consistent with Si–C bonding, verifying the covalent attachment of the organic monolayer to the silicon surface (Fig. 2b). The main peak at 98.2 eV showed the Si itself, while the other two peaks appeared due to rapid oxide formation on the surface. Furthermore, the Pt 4f region displayed characteristic peaks at ~ 71.2 eV and ~ 74.5 eV, confirming the presence of metallic platinum (Fig. 2c). The high-resolution C 1 s spectrum of the Si/Pt + OD sample revealed a dominant peak at 284.8 eV, corresponding to C–C and C–H bonds, indicative of the long alkyl chains in the octadecyl SAM (Fig. 2d). C-Si bonding formation was confirmed by a peak at 282.9 eV, whereas a small peak showing the presence of C-O bonding occurred due to surface contamination. The hybrid “island-and-sea” structure is confirmed, where Pt nanoparticles act as catalytic islands while the SAM forms a continuous hydrophobic sea that protects the underlying silicon.

**Fig. 3:**
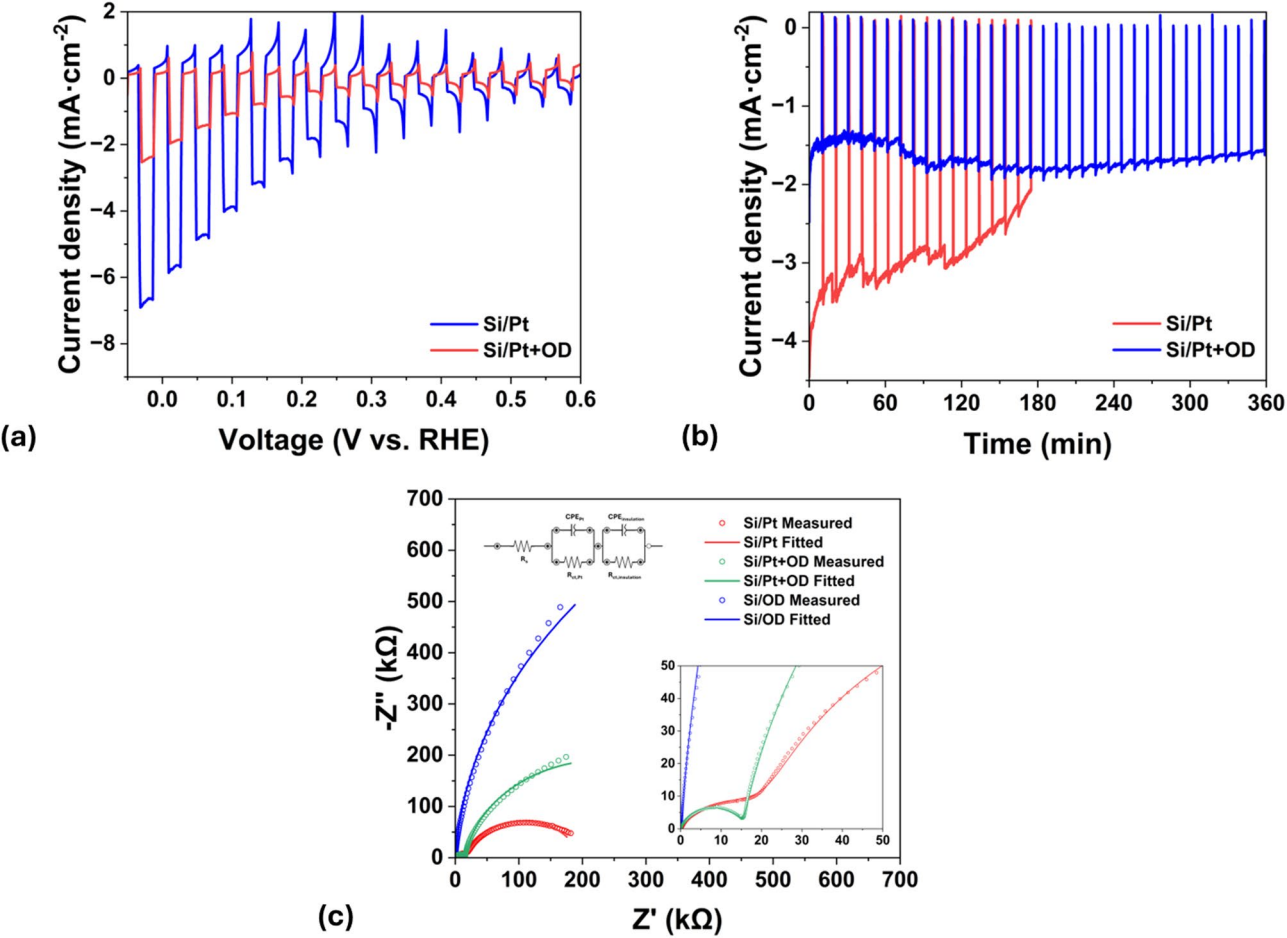
(**a**) PEC performance of Si/Pt and Si/Pt + OD photoelectrodes and (**b**) their stability at 0 V vs. RHE. (**c**) EIS plot of Si/Pt, Si/Pt + OD, and Si/OD photoelectrodes with the equivalent circuit for the system.

Photoelectrochemical tests were conducted to evaluate the effect of octadecyl SAM on the water splitting efficiency and stability. The linear sweep voltammetry test results show that the Si/Pt and Si/Pt + OD are photoactive at 0 V vs. RHE in neutral 0.5 M Na_2_SO_4_ solution (Fig. 3a). Si/Pt shows substantially higher photocurrent compared to Si/Pt + OD. This is attributed to the reduction of surface area that is in contact with the electrolyte and can participate in the hydrogen evolution or corrosion reactions. In this sense, in Si/Pt, the surface is totally exposed to the electrolyte, whereas in Si/Pt + OD, almost all surface area available for reaction is the Pt nanoparticle surface area.

Chronoamperometric test at 0 V vs. RHE was conducted to compare the stability of Si/Pt and Si/Pt + OD. Figure 3b shows that the photocurrent of Si/Pt decreases from 4.0 mA cm^− 2^ to 3.2 mA cm^− 2^ in 1 h and further decreases to 2.0 mA cm^− 2^ in 3 h. In contrast, the photocurrent of Si/Pt + OD does not undergo any major decline throughout the 6-hour stability test in neutral 0.5 M Na_2_SO_4_ electrolyte. Additionally, the PEC performance for bare Si and cyclic voltammetry test was performed and presented in Figure S2.

Furthermore, hydrogen gas produced accumulates on the surface of the photocathode because of the hydrophobic nature of the octadecyl SAM. This further stabilizes the surface by preventing O_2_ from reaching the Si-C bond and by filling the space in the octadecyl SAM between the Si and the electrolyte.

EIS analysis was conducted to gain a deeper understanding of the process on the surface of the electrode. For that, degenerately doped Si (0.001 Ohm cm^− 2^) was used to prepare c-Si/Pt, c-Si/OD, and c-Si/Pt + OD samples. As can be seen in Fig. 3c, all three EIS results can be modelled with the circuit given in the inset. In the circuit, R_s_ depicts the resistivity associated with the electrolyte, which is in the range of 100–124 Ohms in all three cases. The parallel combination of R_ct_,_Pt_ and CPE_Pt_ is related to the presence of Pt nanoparticles on the surface. The R_ct_,_Pt_ retains close values in both c-Si/Pt and c-Si/Pt + OD cases (~ 2E + 4 Ohm). In the case of c-Si/OD, the measured points are fitted to the circuit, where the parameters of R_ct_,_Pt_ and CPE_Pt_ are equal to zero. In the samples c-Si/Pt, the areas not between the Pt particles are covered with a couple of nanometers of native SiO_2_. Therefore, R_ct_,_insulation_ and CPE_insulation_ are related to the resistances of Si/OD and Si/SiO_2_ (native) layers in c-Si/OD and c-Si/Pt, respectively. When comparing R_ct_,_insulation_, it is higher in the case of Si/OD (1.7E + 6 Ohm). Further detailed equivalent circuit fittings with all the values are presented in Figure S3.

**Table 1:** Summary of the recent progress in the stable PEC operation under various pH media.

Photocathode	Protection layer	Electrolyte solution	J_ini_^a^	J_fin_^b^	%_red_^c^	Stability^d^	Ref.
pn^+^-Si/Pt	W-TMOS	1 M HClO_4_ (pH ~ 0)	−35	−35	0%	110 h	^13^
Pt/PADs/Si	SiNx	0.5 M H_2_SO_4_ (pH ~ 0)	−31	−31	0%	100 h	^22^
p-Si	n-SnO_2_/n-Fe_2_O_3_	0.25 M Na_2_SO_4_ with Phosphate Buffered Saline (pH ~ 7.25)	−0.3	−1	−233%	2.5 h^e^	^23^
p-Si/TiO_2_	NiO_x_	0.2 M Potassium phosphate buffer (pH ~ 7)	−1.48	−1.48	0%	5 h	^24^
Pt/InAs NW/p-Si	TiO_2_	0.5 M Na_2_SO_4_ with Phosphate buffer (pH ~ 7)	−1	−0.8	20%	20 h	^25^
Si	TiO_2_/Co(CR-DCP)	0.1 M acetate buffer (pH ~ 4.5)	−0.527	−0.527	0%	10 h	^26^
Si/Pt	OD	0.5 M Na_2_SO_4_ (pH ~ 7)	−1.5	−1.5	0%	6 h	This work

[a] Initial photocurrent density (mA cm^-2^) at the beginning of the stability test. [b] Final photocurrent density (mA cm^-2^) at the end of the stability test. [c] Percentage of reduction in the current density of photoelectrodes, (J_ini_-J_fin_)/J_ini_ x 100. [d] Duration between the beginning and end of the stability test, all performed at 0 V vs. RHE, except those indicated in the table. [e] Stability test performed at -0.33 V vs. RHE.

Although Si is considered a promising photoelectrode, it suffers from corrosion in both acidic and neutral environments. Li et al.^27^ showed that Si/Pt degrades severely in the acidic electrolyte within 2 h, which is correlated with the degradation trend of the Si/Pt sample in our work. To further show the relevance of this report, Table 1 summarises recent progress in stabilising Si photocathodes across various pH conditions. Notably, high-performance photocathodes passivated with stable protection layers have been developed for PEC operation in strongly acidic environments (pH ~ 0–1)^13,22^. However, these systems are not compatible with neutral medium operation.

In neutral environments, the photocurrent density of Si-based electrodes remains limited (≤ − 1.5 mA cm^− 2^), and stable operation is observed during short-term operation (≤ 20 h), while mainly using TiO_2_-based passivation layers^23–26^. Even though TiO_2_ and other inorganic protection layers offer chemical stability, their deposition usually requires expensive and non-scalable techniques such as plasma-enhanced ALD^25^. For Si-based photocathodes, only one study has reported a stable organic protective layer, W-TMOS, considered as an organosilane, operating for over 110 h in acidic electrolyte (pH ~ 0)^13^. However, there are no more groundbreaking studies on organic passivation of Si for neutral-pH PEC operation.

In this context, our Si/Pt photocathode, protected by a solution-processable 1-octadecyl layer, exhibits enhanced stability under neutral conditions, maintaining a stable photocurrent of −1.5 mA cm^− 2^ with negligible degradation over 6 h. This result provides evidence of stable organic passivation for Si photocathodes operating under neutral conditions. The simplicity and scalability of this concept may represent a promising direction toward practical and sustainable PEC hydrogen production.

However, to critically evaluate this work, it is important to mention several limitations that occur despite the enhanced performance demonstrated. The long-term performance evaluation was performed in a benign Na_2_SO_4_ electrolyte (pH ~ 7) rather than natural seawater. For example, Zhao et al.^28^ prepared a substitute alkaline seawater following the Standard Practice for the Preparation of Substitute Ocean Water due to the risk of additional corrosion and side-reaction pathways caused by chlorides and other ions present in real ocean water. Additionally, to further evaluate degradation pathways, which are not always clear under constant illumination, it would be crucial to conduct extended on/off cycling tests (12 h/12 h) as presented in prior studies^29,30^. Tackling these limitations will be important future work for advancing the stability of photoelectrodes for solar water splitting.

## Conclusion

In this study, we demonstrated an “island-and-sea” strategy for enhancing the stability of silicon-based photoelectrodes for neutral-pH PEC water splitting. By decorating pn-Si surfaces with Pt nanoparticles and passivating the remaining surface with a hydrophobic 1-octadecyl SAM, we enhanced both charge transfer efficiency and corrosion protection performance. The Pt nanoparticles serve as catalytic sites for the HER, while the SAM prevents direct contact between the silicon surface and the electrolyte. PEC measurements showed that the Si/Pt + OD electrodes maintained a stable photocurrent over 6 h of operation in neutral electrolyte, whereas bare Si/Pt electrodes exhibited rapid degradation. This approach circumvents the limitations of traditional oxide-based protective layers, such as the complexity of fabrication and alteration of the band structure. Our findings suggest that hybrid organic-inorganic surface engineering can be extended to other unstable photoelectrode materials and further used to improve the long-term stability of neutral-pH water splitting systems.

## Methods

### Pt decoration of pn-Si

Commercially available monocrystalline p-n junction solar Si with pyramidal morphology was used. The thickness of the n layer is 350 nm on a 500 micron p-Si crystal, and it has an Al back contact. The wafer was cut into 1 × 1 cm pieces, and the anti-reflective coating was removed with HF. Pt nanoparticle electrocatalyst was deposited on the surface of the p-n-Si by the electroless deposition method described by Yin et al.^9^. Briefly, the pn-Si samples were cleaned in acetone, methanol, and via O_2_ plasma cleaning. After insulating the back contact with Kapton tape, the native oxide layer on the surface was removed by immersing the wafer in 5% HF for 1 min. Pt nanoparticles were deposited in an isopropanol solution containing 5% water, 2 M HF, and 1 mM H_2_PtCl_6_·6H_2_O by immersing for 4 min. After Pt deposition, the samples were washed with DI water.

### 1-octadecyl SAM deposition

1-octadecyl SAM was deposited on the surface of pn-Si by following the procedure reported by Bhairamadgi et al.^21^. After electroless Pt deposition, the sample was immediately immersed in N_2_ bubbled 1-octadecene. This was done to prevent native SiO_2_ formation on the surface of Si, which will reduce the quality of the SAM. After that, the sample was left in the OD under a 254 nm wavelength UV lamp for 24 h. After the reaction was over, the samples were washed with acetone and dried.

### Structural characterization

Contact angle measurements were initially performed on a goniometer (OSA-15EC) to examine the hydrophobicity of highly conductive Si samples (c-Si) covered by an OD SAM. The samples were then investigated using a Scanning Electron Microscope (SEM, ZEISS Crossbeam 540) to confirm the deposition of Pt nanoparticles. An X-ray photoelectron Spectrometer (XPS, NEXSA (Thermo Scientific)) was used for the elemental analysis to evaluate the formation of “island-and-sea” on the surface of c-Si.

### Photoelectrochemical tests

The photoelectrochemical (PEC) measurements were conducted using a PalmSens4 Potentiostat in an ordinary three-electrode cell configuration by using samples as the photocathode, a Pt coil as a counter electrode, and an Ag/AgCl electrode as the reference electrode. The water splitting photocurrent was measured with 0.5 M Na_2_SO_4_ (pH ≈ 7) aqueous solution at a scan rate of 2 mV·s^− 1^. The cell was illuminated with simulated AM 1.5 light by a Newport TLS260-300X Xenon lamp solar simulator, and the light power density was adjusted by a silicon solar cell to 100 mW·cm^− 2^. The potentials in the collected data were converted to the RHE by the equation:

$$\:{E}_{RHE}={E}_{Ag/AgCl}+0.197V+0.059\times\:pH$$

EIS analysis was performed to better understand the effect of octadecyl SAM on the charge transfer. Degenerative doped c-Si samples were used to prepare c-Si/Pt, c-Si/OD, and c-Si/Pt + OD through the same method to prepare the pn-Si samples. EIS analysis was performed in a 0.5 M Na_2_SO_4_ electrolyte with a PalmSens4 Potentiostat.

## Supplementary Information

Below is the link to the electronic supplementary material.

Supplementary Material 1

## Data Availability

The data supporting the findings of this research are available within the article and supplementary information.
