# Supplementary material for: Hybrid island-and-sea approach for corrosion protection of Si photocathode in neutral-pH water splitting
Source: Sci Rep. 2025 Dec 3;16:1064. doi: 10.1038/s41598-025-30589-y (PMC12783614; doi:10.1038/s41598-025-30589-y)
Supplement: Supplementary file 1 — Supplementary Material 1 [file 41598_2025_30589_MOESM1_ESM.docx]

**Supporting Information for**

**Hybrid Island-and-Sea Approach for Corrosion Protection of Si Photocathode in Neutral-pH Water Splitting**

Magzhan Amze^1^, Asset Aliyev^1^, Yerbolat Magazov^1^, Nurxat Nuraje^1,2^, Kadyrzhan Dikhanbayev^3^ and Erkin Shabdan^1,4,*^

^1^Renewable Energy Lab, National Laboratory Astana, Nazarbayev University, Astana, 010000, Kazakhstan

^2^Department of Chemical and Materials Engineering, School of Engineering and Digital Sciences, Nazarbayev University, Astana, 010000, Kazakhstan

^3^Faculty of Physics and Technology, Al-Farabi Kazakh National University, Almaty, 050040, Kazakhstan

^4^School of Intelligent Systems, Astana IT University, Astana, 010000, Kazakhstan

^*^Corresponding author [yerkin.shabdan@nu.edu.kz](mailto:yerkin.shabdan@nu.edu.kz);

**Table of Contents**

S1. SEM images of the Si/Pt photoelectrode.

S2. Photoelectrochemical tests.

S3. Equivalent circuit fitting.

**S1. SEM images of the Si/Pt photoelectrode**


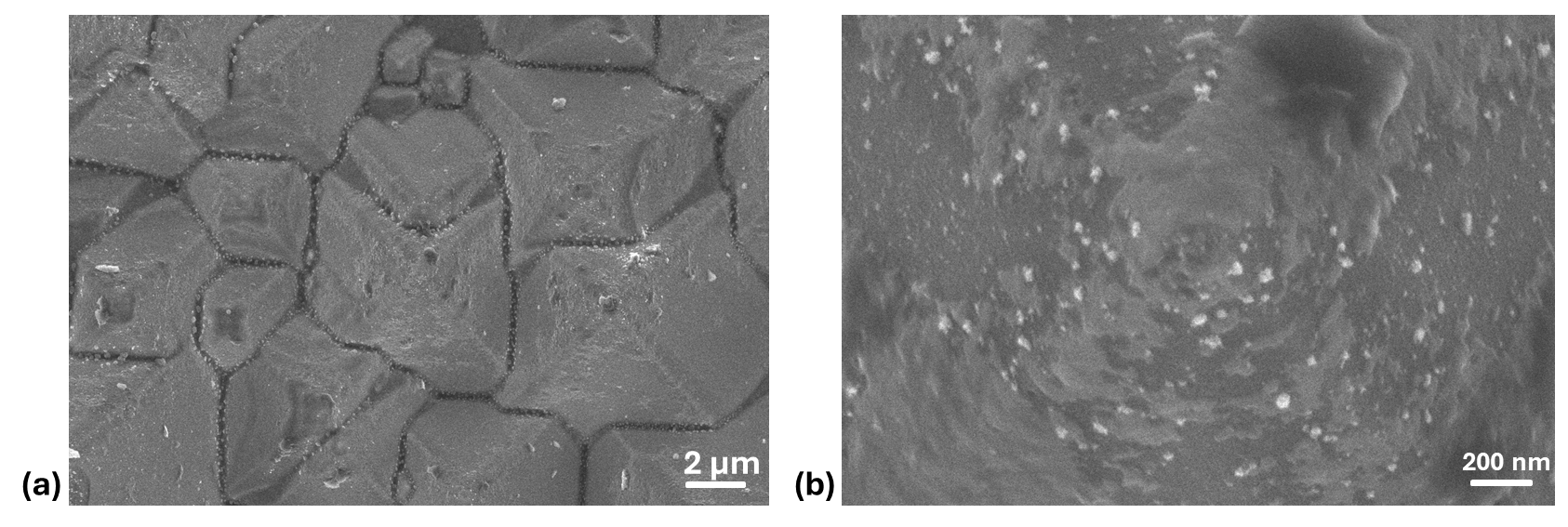


**Figure S1.** SEM images of the Si/Pt photoelectrode

**S2. Photoelectrochemical tests**


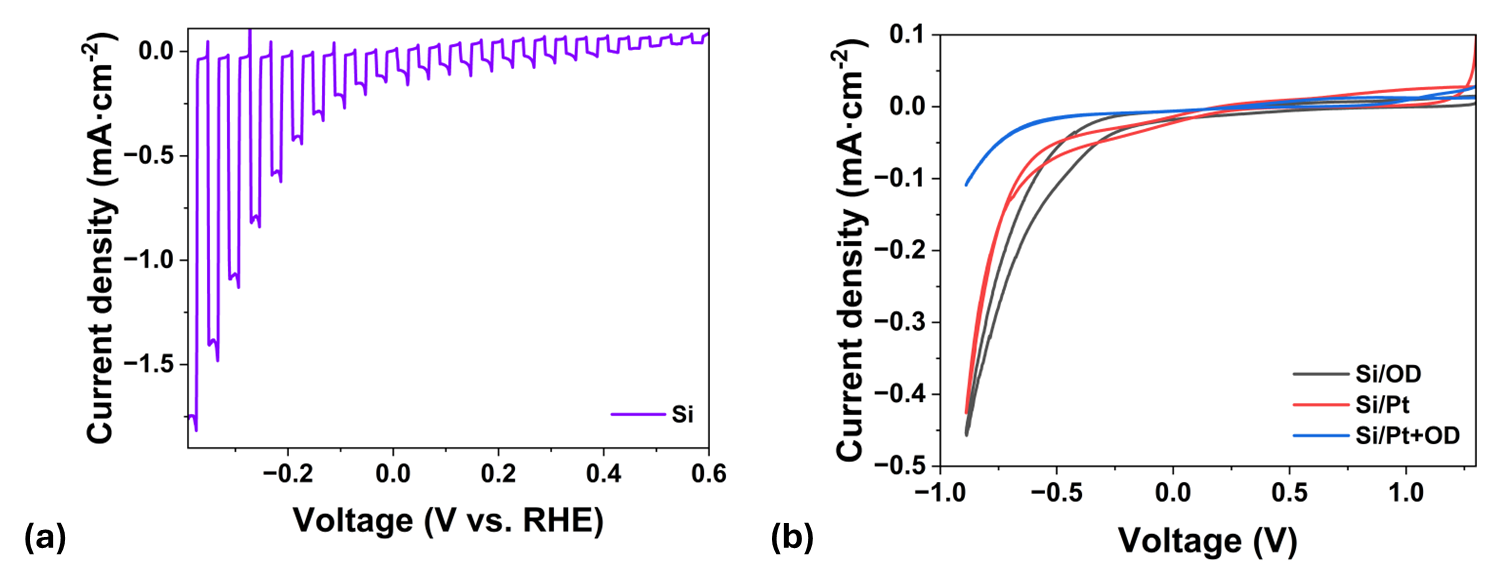


**Figure S2.** (a) PEC performance of a bare Si. (b) Cyclic voltammetry of fabricated photoelectrodes.

**S3. Equivalent circuit fitting**


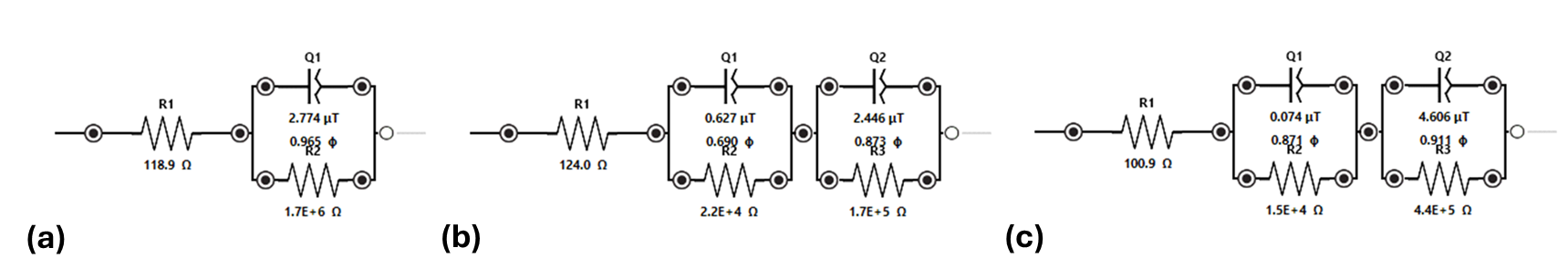


**Figure S3.** Equivalent circuit fitting and detailed values based on the EIS result for (a) Si/OD, (b) Si/Pt, and (c) Si/Pt+OD
